# Supplementary material for: DMRforPairs: identifying Differentially Methylated Regions between unique samples using array based methylation profiles
Source: BMC Bioinformatics. 2014 May 15;15:141. doi: 10.1186/1471-2105-15-141 (PMC4046028; doi:10.1186/1471-2105-15-141)
Supplement: Additional file 2 — DMRforPairs output for the comparison of A431-MCF7 and NA17018-NA17105. Please start from the HTML files in each folder. Available via the BMC Bioinformatics website. [file 1471-2105-15-141-S2.zip › 1394847754114233_MOESM2_ESM/A431_MCF7/figures/27629.pdf]

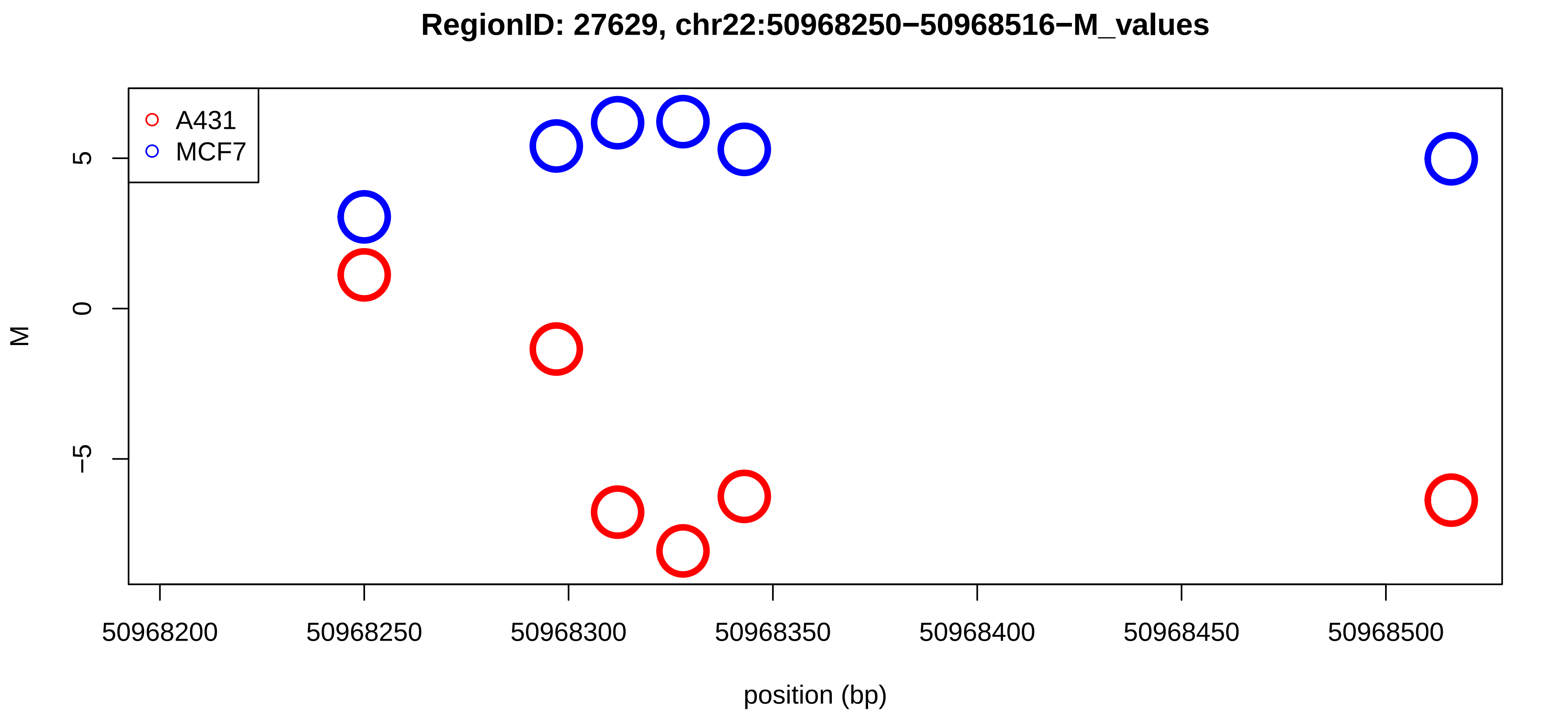
RegionID: 27629, chr22:50968250−50968516−M_values
A431
MCF7
M
5
0
−5
50968200
50968250
50968300
50968350
50968400
50968450
50968500
position (bp)

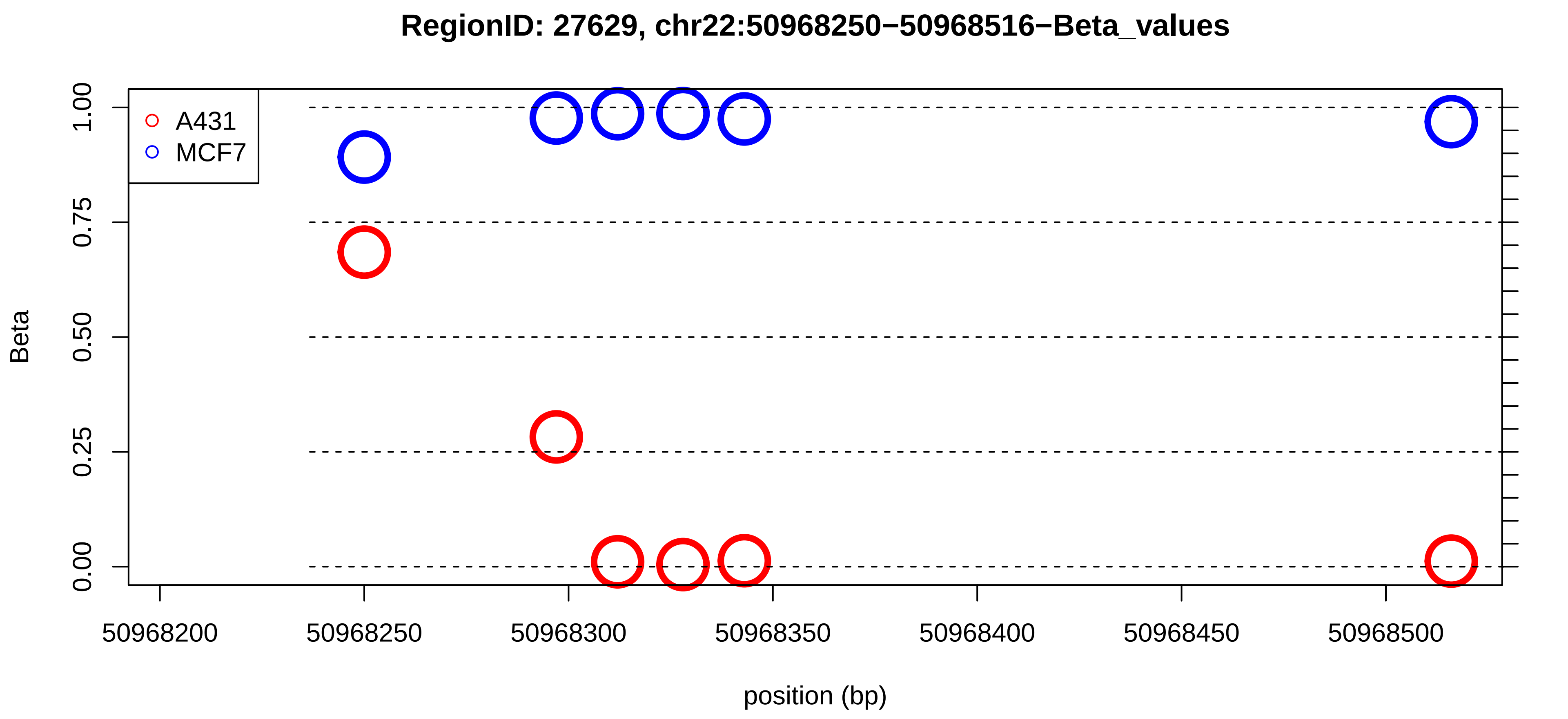
RegionID: 27629, chr22:50968250−50968516−Beta_values
A431
MCF7
Beta
1.00
0.75
0.50
0.25
0.00
50968200
50968250
50968300
50968350
50968400
50968450
50968500
position (bp)

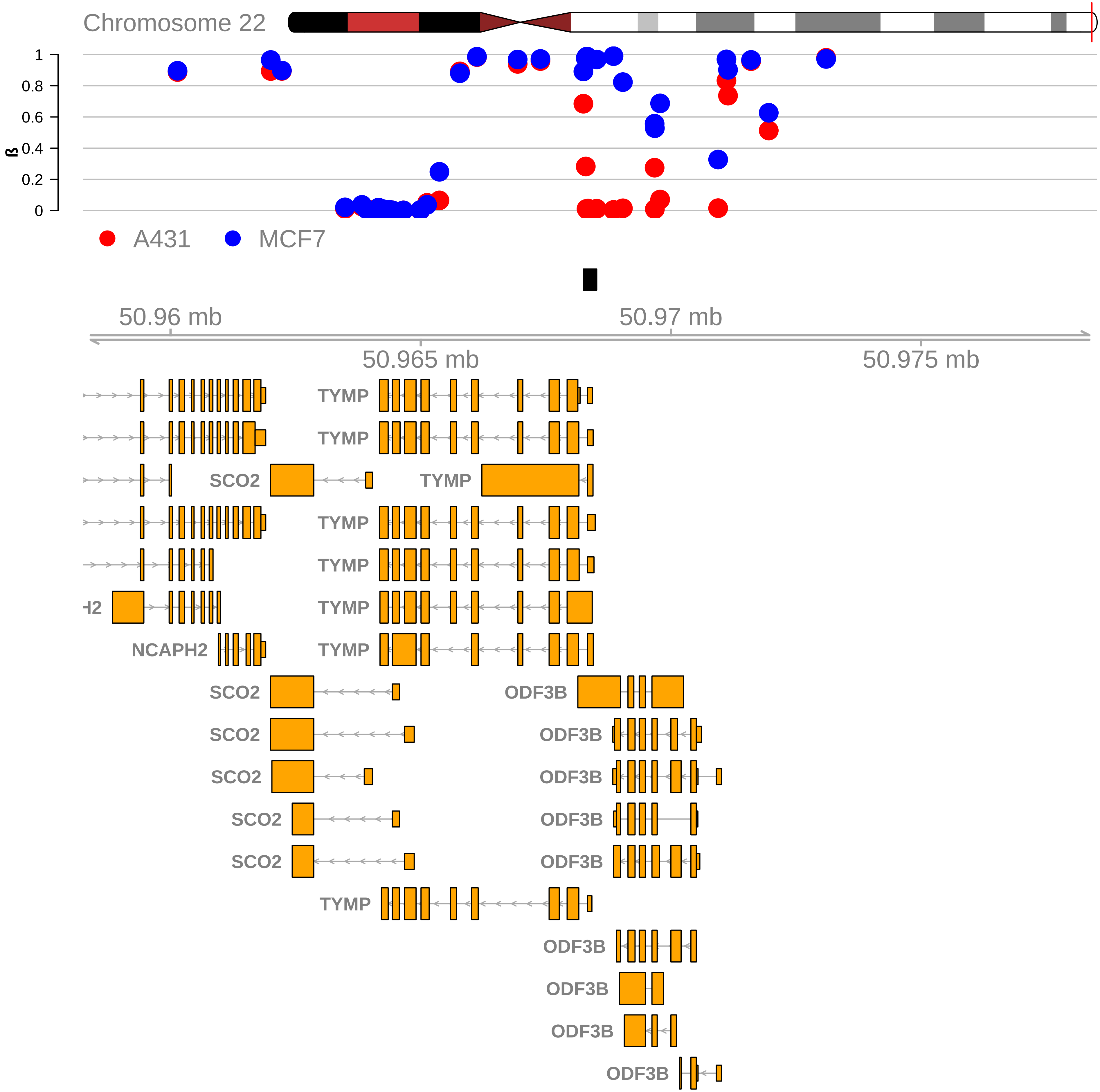

Chromosome 22
1
0.8
0.6
ß 0.4
0.2
0
A431
MCF7
50.96 mb
50.97 mb
50.965 mb
50.975 mb
TYMP
TYMP
SCO2
TYMP
TYMP
TYMP
H2
TYMP
NCAPH2
TYMP
SCO2
ODF3B
SCO2
ODF3B
SCO2
ODF3B
SCO2
ODF3B
SCO2
ODF3B
TYMP
ODF3B
ODF3B
ODF3B
ODF3B
